# The Potential of Mesenchymal Stromal Cells in Neuroblastoma Therapy for Delivery of Anti-Cancer Agents and Hematopoietic Recovery

**DOI:** 10.3390/jpm11030161

**Published:** 2021-02-25

**Authors:** Caroline Hochheuser, Nina Y. Kunze, Godelieve A. M. Tytgat, Carlijn Voermans, Ilse Timmerman

**Affiliations:** 1 Princess Maxima Center for Pediatric Oncology, 3584 CS Utrecht, The Netherlands; c.h.hochheuser@prinsesmaximacentrum.nl (C.H.); g.a.m.tytgat@prinsesmaximacentrum.nl (G.A.M.T.); 2 Sanquin Research and Landsteiner Laboratory, Department of Hematopoiesis, Amsterdam UMC, University of Amsterdam, 1066 CX Amsterdam, The Netherlands; n.kunze@sanquin.nl (N.Y.K.); c.voermans@sanquin.nl (C.V.)

**Keywords:** neuroblastoma, mesenchymal stem/stromal cells, hematopoietic stem cell transplantation, drug delivery, oncolytic virotherapy, biodistribution, cellular therapy

## Abstract

Neuroblastoma is one of the most common pediatric cancers and a major cause of cancer-related death in infancy. Conventional therapies including high-dose chemotherapy, stem cell transplantation, and immunotherapy approach a limit in the treatment of high-risk neuroblastoma and prevention of relapse. In the last two decades, research unraveled a potential use of mesenchymal stromal cells in tumor therapy, as tumor-selective delivery vehicles for therapeutic compounds and oncolytic viruses and by means of supporting hematopoietic stem cell transplantation. Based on pre-clinical and clinical advances in neuroblastoma and other malignancies, we assess both the strong potential and the associated risks of using mesenchymal stromal cells in the therapy for neuroblastoma. Furthermore, we examine feasibility and safety aspects and discuss future directions for harnessing the advantageous properties of mesenchymal stromal cells for the advancement of therapy success.

## 1. Introduction

In the search for new effective tumor treatments, the potential of different forms of cellular therapy is more and more unraveled. Different cells of the immune and hematopoietic system are already used to improve the clinical outcome of cancer patients, including hematopoietic stem cells and T cells engineered to target tumor cells via specific surface molecules [1,2]. Cellular therapy becomes especially relevant in refractory tumors, in which conventional therapies are not effective anymore.

One of these metastatic, frequently relapsing cancers is neuroblastoma (NB). Being the most common extracranial solid childhood tumor, NB accounts for 7–10% of all childhood malignancies [3,4]. NB tumors originate from cells of the neural crest, a tissue that gives rise to the sympathetic nervous system during embryonic development [5]. Accordingly, NB primary tumors mainly manifest in the adrenal glands and sympathetic ganglia, are highly heterogeneous and occur at a young age [6]. In fact, NB is the most commonly diagnosed tumor in children under one year of age [7]. About half of NB patients present with metastasis at diagnosis [8] with bone marrow (BM) involvement in 90% of cases [9]. Corresponding to the heterogeneity of NB tumors, the International Neuroblastoma Risk Group (INRG) classification system categorizes patients into very low-, low-, intermediate- and high-risk groups, based on a set of prognostic markers and five-year event-free survival rates [10]. Next to dissemination status, high-risk prognostic markers include advanced age (>18 months), *MYCN* amplification, and chromosome 11q aberration. Non-high-risk patients receive a moderate treatment, including surgery and/or chemotherapy [11,12], which results in survival rates of more than 90% [13]. The survival rate of high-risk patients, however, remains below 50% [10], and 50–60% of these patients experience relapse [6] despite intense multi-modal treatment, comprising induction chemotherapy to induce remission, resection of the primary tumor, myeloablative therapy (MAT) with autologous hematopoietic stem cell transplantation (ASCT) and radiation therapy, in addition to post-consolidative immunotherapy accompanied by the differentiation agent isotretinoin [10]. For ASCT, the patient’s hematopoietic stem- and progenitor cells (HSPCs) are mobilized into the peripheral blood (PB), collected via apheresis, and later reinfused into the patient’s bloodstream in order to reconstitute the hematopoietic system after MAT. With ASCT as the standard of care, the efficacy of a single or double transplant, as described by Park et al. [14], is at present investigated in the HR-NBL2 trial (NCT04221035) of the International Society of Paediatric Oncology-Europa-Neuroblastoma (SIOPEN). Because NB is a major cause of cancer-related death in infancy and considering the, sometimes severe, side effects of conventional treatment [15], there is a demand for research on alternative treatment strategies to combat NB.

One promising candidate for cellular therapy in NB patients is multipotent mesenchymal stromal cell (MSC). The abbreviation “MSCs” has been used for mesenchymal stem cells in the past but is nowadays used as a broader term to also include cells whose biologic characteristics do not meet the definition of stem cells [16,17]. Here, we use the term MSCs to describe multipotent mesenchymal stromal cells, for which the International Society for Cellular Therapy (ISCT) suggested the following minimal definition criteria: (i) expression of CD105, CD73, and CD90, and lack of expression of CD45, CD34, CD14 or CD11b, CD79a, or CD19 and human leukocyte antigen (HLA)-DR surface molecules; (ii) potential to differentiate into osteoblasts, adipocytes, and chondroblasts; and (iii) adherence to plastic in standard culture conditions [18]. MSCs are present in many tissues, including bone marrow (BM), adipose tissue (AT), umbilical cord (UC), dental pulp, and placenta [19]. The source of MSCs influences their phenotype, differentiation and migration potential, and immunomodulatory capacity [20,21,22,23] and is therefore important to consider when applying MSCs in cellular therapy. The characteristics and (dis-) advantages for clinical applications of MSCs from frequently used sources (mainly AT- and BM-derived MSCs, in the following written as AT–MSCs and BM–MSCs, respectively) have been reviewed elsewhere [24,25]. 

BM–MSCs are of special interest since their native environment presents the primary or metastatic site for various (hematologic) malignancies [26]. In the healthy BM, MSCs and other stromal cells such as endothelial cells, osteoblasts, and C-X-C motif chemokine ligand 12 (CXCL12)-abundant reticular (CAR) cells in the perivascular and endosteal niche play an important role in maintaining the balance of self-renewal and differentiation of HSPCs [27,28,29]. MSCs contribute to the BM hematopoietic niche through cell–cell interactions and secreted factors (e.g., CXCL12 [29], stem cell factor (SCF/Kit-ligand) [30], Wnt signaling components [31], thrombopoietin (TPO) [32], angiopoietin (Ang-1) [33] and interleukin (IL)-7; reviewed by Ehninger et al., 2011 [28]), and by differentiating into various other cell types, including adipocytes and osteoblasts [34]. In addition to the ability to support the hematopoietic system, the most prominent functions of MSCs in cellular tumor therapy are their immunomodulatory function [35] and their ability to sense inflammation, which allows them to migrate to damaged tissues, including sites of tumor growth [36]. It is important to note that the function of MSCs can be altered in the disease context, for example when being influenced by cancer cells to support the tumor [37,38,39,40,41,42,43]. In this light, there is a scientific controversy whether MSCs act tumor-supportive or -suppressive in physiological conditions (reviewed recently by our group [44] in NB context, suggesting a primarily tumor-supportive effect) and whether they bear a risk to contribute to disease progression when used in cellular therapy [45]. 

This review aims to evaluate the potential use of MSCs in therapy for NB. It outlines results obtained both in animal models and in clinical trials studying the use of MSCs as delivery vehicles for anti-cancer agents and for supporting hematopoietic stem cell transplantation (HSCT) after MAT. Important factors influencing the efficacy of these treatments will be discussed, including the biodistribution of systemically applied MSCs and the risk of an adverse effect on tumor progression. Finally, existing knowledge gaps will be summarized in combination with an assessment of possible future directions.

## 2. MSCs as Delivery Vehicles

**Figure 1:**
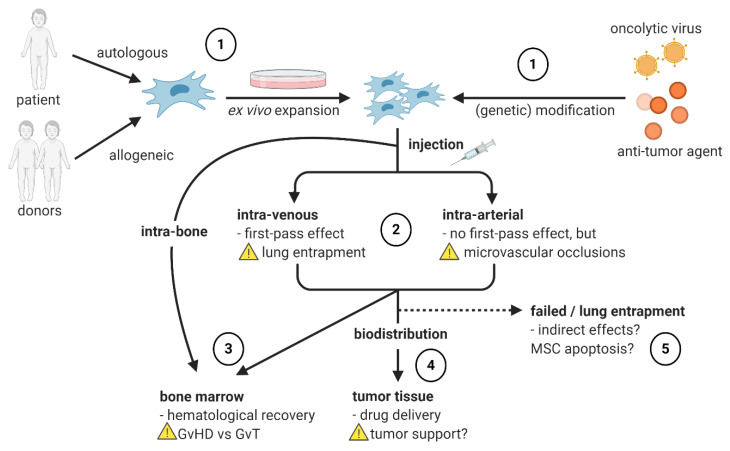
Overview of procedures and potential risks in therapeutic MSC applications. ① MSCs derived from the patient (autologous) or a donor (allogeneic) are culturally expanded, optionally equipped with an anti-cancer agent or oncolytic virus, and injected into the patient. ② The systemic application can entail lung entrapment (intravenous application) or microvascular occlusions (intra-arterial application). ③MSC homing to the bone marrow can also be achieved by intra-bone injection. The purpose of MSCs in the BM niche is to support hematologic recovery and prevent graft-versus-host disease (GvHD) in case of an allogeneic transplant, but they can at the same time diminish a desirable graft-versus-tumor (GvT) effect. ④ Inflammation-sensing properties allow MSCs to migrate and deliver drugs to the tumor tissue but bear the risk of tumor support. ⑤ Even when MSCs get entrapped in the lungs or fail to reach their target tissue for other reasons, their expected effects can often be observed nevertheless. Indirect effects, e.g., signals of apoptotic MSCs to immune cells [48], could be responsible for that.

To lower the chemotherapeutic burden and reduce side effects for patients, a new addition to classical NB treatment might be the tumor-targeted delivery of anti-cancer agents and oncolytic viruses (Figure 1 ①)—the inflammation-sensing nature of MSCs allows effective recruitment of modified MSCs to tumor sites and potentially even to micro-metastases, a common source of relapse. Additionally, due to a lack of MHC recognition patterns, MSCs are immunologically inert, allowing the use of allogeneic cells [46]. This locally acting therapy has therefore the potential to be less toxic than systemically applied conventional chemotherapy. In the context of NB, MSC-delivered chemotherapeutic agents (paclitaxel), soluble factors with an anti-cancer effect (TNF-related apoptosis-inducing ligand (TRAIL), IFN-β, IFN-γ, IL2), microRNAs (miR-124), and oncolytic viruses have been studied so far. While the overall aim of tumor-targeted delivery is a strong localized anti-tumor effect, there are additional advantages of MSCs as the delivering units which are further described in the following section, e.g., their immunomodulatory effects (especially relevant for oncolytic virus delivery) or a sustained supply of the anti-cancer agents through stable expression by MSCs. Importantly, treatment resistance, which presents a major obstacle for example in anti-cancer therapy with TRAIL, can be overcome when using MSCs as delivery vehicles [47].

### 2.1. MSCs Delivering Anti-Cancer Agents

One of the anti-cancer agents investigated in this context is TNF-related apoptosis-inducing ligand (TRAIL), which causes apoptosis primarily in tumor cells by binding to its cognate death receptors (DR4 and DR5) [49]. While the soluble, truncated form of TRAIL has a short half-life in the bloodstream and bears the risk of inducing resistance, MSCs as delivery vehicles allow a continuous expression of the full-length form of TRAIL at the tumor site. A recent pre-clinical study showed the ability of TRAIL-expressing MSCs to kill classical and primary NB cell lines in vitro and to successfully migrate to tumor sites in vivo, although a reduction of NB tumor growth in xenotransplantation experiments could only be reached in combination with the anti-cancer drug bortezomib [46]. Interestingly, researchers artificially increased the tumor-tropism of TRAIL-expressing MSCs toward GD2-positive glioblastoma by equipping them with a truncated anti-GD2 chimeric antigen receptor (CAR) [50], a strategy that has similarly been used for selectively targeting CAR T cells to GD2-expressing NB cells [51]. Another approach for TRAIL delivery utilized exosomes derived from TRAIL-engineered murine BM–MSCs, which significantly reduced tumor volume and induced necrosis in a melanoma mouse model [52].

Other approaches make use of engineered interferon (IFN)-expressing MSCs. IFN-β was one of the first anti-cancer agents to be engineered into BM–MSCs as drug delivery vehicles, which subsequently decreased tumor growth in vivo [53]. The potential of IFN-β to inhibit tumor growth is mediated by immunostimulatory [54], antiangiogenic [55], and antiproliferative effects [56]. In NB, the survival of tumor-bearing mice was significantly increased when IFN-β-expressing murine BM–MSCs were delivered intraperitoneally [57]. Similarly, IFN-γ is known to decrease tumor proliferation and neoangiogenesis [58], but systemic application in clinical trials failed due to associated toxicities [59]. Research using IFN-γ-expressing BM–MSCs as delivery vehicles demonstrated decreased tumor growth and increased overall survival of NB tumor-bearing mice after intratumoral injection [59]. The effect was shown to be mediated by polarizing host macrophages into the pro-inflammatory M1 phenotype. However, increased IFN-γ expression was shown to impair the hematopoietic support function of BM–MSCs in mouse models [60], suggesting that this therapy could have unfavorable side effects on the already impaired BM of high-risk NB patients. While these studies do show the effectiveness of anti-cancer agents when being delivered by MSCs, the reproducibility in humans is not ascertained because one of the most prevalent clinical administration routes is intravenous instead of intraperitoneal or intratumoral [61].

Other promising results were obtained by researchers who previously proved an anti-cancer effect of BM– and AT–MSCs loaded with the chemotherapeutical compound paclitaxel [62]. In order to overcome various translational limitations of their previous model, such as high good manufacturing practice (GMP) standard and limited drug concentration at the tumor site, their following study utilized paclitaxel-loaded micro-fragmented adipose tissue (MFAT), which has a high content of MSCs [63]. Implanted next to the primary tumor site after surgical resection in an NB mouse model, MFAT functioned as a scaffold containing MSCs that released the drug over a prolonged period of time, preventing NB relapse. Similarly, delivery of paclitaxel in BM–MSC-derived exosomes demonstrated successful homing, penetration, and anti-tumor efficacy in a mouse model of pancreatic cancer [64].

Furthermore, recent in vitro work indicated the potential use of a nervous system-specific microRNA, miR-124, in order to induce differentiation in NB cells [65]. Co-culture experiments with miR-124-expressing AT–MSCs indeed demonstrated decreased proliferation and increased apoptosis as well as differentiation of NB cells. The same effect was observed with exosomes derived from these MSCs, opening new interesting research lines of a cell-free treatment approach (also discussed below in the chapter “Cell-Free Approach Using Extracellular Vesicles”).

Another approach using MSCs as delivery vehicles is the engineered expression of IL2, a cytokine frequently used in cancer therapy due to its stimulatory effect on CD8+ T cells and NK cells [66]. Interestingly, an in vitro study with AT–MSCs engineered to express IL2 described opposing effects of these MSCs depending on culture method [67]: While in direct co-culture, a reduction in NB cell proliferation was observed, the opposite effect (increased proliferation) was the case when culturing NB cells in conditioned medium of IL2-expressing MSCs. Furthermore, induced expression of IL2 in MSCs concomitantly upregulated transcription and protein expression of pro-tumorigenic factors such as MMP2 and TGF-β1 in MSCs. 

There are currently no clinical trials investigating the use of MSCs for the delivery of therapeutic agents in NB patients. Clinical trials in the context of other tumors are listed in Table S1A–C. Results from these studies are not (yet) available.

### 2.2. MSCs Delivering Oncolytic Viruses

Next to the above-mentioned anti-cancer agents, MSCs are also used as delivery vehicles for oncolytic viruses. The latter are virus strains that are either naturally non-virulent in humans or genetically engineered to ensure that they replicate selectively in tumor cells (reviewed by Fukuhara et al. [68]). This strategy has several advantages in addition to the targeting effect. Firstly, the immunosuppressive properties of MSCs facilitate a balanced immune response, which is crucial for the success of oncolytic virotherapy: A too strong “anti-viral” immune response needs to be avoided in order for the virus to lyse the tumor cells efficiently. After lysis, tumor-specific antigens are released and provoke an “anti-tumor” immune response [69]. The immunomodulatory function of MSCs could thus help to delay the immune response against the virus sufficiently, allowing a decent anti-tumor response. Secondly, in contrast to systemic injection of oncolytic virus, the use of MSCs hides the virus from the immune system before it becomes active and causes a local inflammatory response despite the immunosuppressive environment created by the tumor. This non-systemic immune response allows the application of several treatment rounds [70]. 

Oncolytic adenovirus-infected MSCs have a viability window of 48–72 h after infection [71] and studies in ovarian cancer have shown that this time period is sufficient to reach the tumor site [72]. The feasibility of oncolytic virus-infected MSC products has been proven pre-clinically [73] and showed only small, self-limiting side effects, such as fever, chills, and discomfort in exploratory studies in NB patients [71,74] (Table 1A,B). In the first study, multi-dose systemic infusion of irradiated oncolytic virus-loaded MSCs (“CELYVIR”) led to complete response (CR) in one out of four patients and even reduced metastatic lesions in the BM of that patient [71]. In the second study, 5 out of 12 patients achieved a positive clinical response (either complete, partial, or stabilization) [74]. Interestingly, in both studies, MSCs had been irradiated before transfusion into the patient in order to avoid tumor-supportive effects of MSCs.

Further beneficial effects of the CELYVIR treatment in NB therapy were proven in a phase I/II clinical trial of adult and pediatric patients with a variety of relapsed/refractory tumors [70] (Table 1C). After intravenous application of CELYVIR, two out of four NB patients in this study showed disease stabilization. Furthermore, persistent detection of viral RNA in the blood of most pediatric patients indicated a successful delivery and replication of the virus at the tumor site. An increasingly higher number of circulating lymphocytes was found in patients that responded to the therapy, suggesting the importance of an active immune system [70]. Another study with CELYVIR identified the T-cell count of patients prior to therapy, a lower pro-inflammatory profile (including lower expression of IFN-γ, IL6, IL8, IDO, and VEGFα) and expression of adhesion molecules (CXCR1, CCR1) on MSCs as factors predicting response to the therapy [74]. Furthermore, immune infiltration and T-cell diversity are suggested to play an important role [75]. Interestingly, a study comparing the effect of CELYVIR with syngeneic and allogeneic MSCs in mouse models found a similar effect of both, proposing a potential use of donor-derived MSCs for oncolytic virus delivery in patients in future clinical trials [76].

Two other clinical trials studying oncolytic virus delivery by MSCs in the context of other tumor types are listed in Table S1D,E.

## 3. MSCs in Hematopoietic Stem Cell Transplantation

High-dose chemotherapy is an important part of the standard-of-care treatment for high-risk NB patients because it allows efficient elimination of tumor cells and thus reduces the risk of relapse caused by minimal residual disease (MRD) [10]. To ensure hematopoietic recovery after MAT, HSPCs are reinfused after the chemotherapy by means of ASCT [77]. MSCs might have the potential to provide an overall functional BM niche after MAT by supporting the engraftment of HSPCs in the BM hematopoietic niche and repairing the damaged tissue [78], (Figure 1 ③). Enhanced in vivo HSPC long-term engraftment facilitated by MSCs was first demonstrated in mouse models [79], and this supportive potential is at present investigated in multiple clinical trials (Table 2). Here, a distinction must be made between the use of either autologous, allogeneic, or third-party MSCs to support the engraftment of either autologous or allogeneic HSPCs. Furthermore, it is important to note that third-party MSCs can also be infused at a later time to treat graft-versus-host disease (GvHD) after allogeneic HSCT [80,81].

### 3.1. Allogeneic MSCs

By far the most frequent application of MSCs during HSCT is that of “allogeneic” MSCs. In the context of ASCT, allogeneic MSCs have been co-infused to treat HSPC engraftment failure. Both a case report of acute myeloid leukemia (AML) patient with incomplete engraftment and BM failure [84] and a phase II clinical trial in patients with hematologic malignancies [85] have demonstrated successful hematopoietic reconstitution after infusion of allogeneic MSCs, defined by neutrophil-, granulocyte-, and platelet recovery (Table S2A,B). 

Allogeneic MSCs are predominantly used during allogeneic HSCT in the treatment of various malignancies. Of note, in the case of NB, allogeneic HSCT is only investigated for relapsed patients, with the desired graft-versus-tumor (GvT) effect as a possible indication [86], and is thus far less frequently applied than ASCT [87]. Possible complications of allogeneic HSCT are induction of GvHD due to donor T-cell reactivity against the recipient and insufficient engraftment of HSPCs, resulting in the delayed recovery of the hematopoietic system. MSCs can be co-transplanted in order to (i) alleviate the risk of GvHD due to their immunomodulatory functions and (ii) facilitate donor HSPC engraftment [88]. A single-arm study by Lazarus et al. in 46 patients with hematologic malignancies proved the safety and feasibility of such allogeneic MSC co-transplantation [89].

In NB, clinical trials investigating HLA-mismatched haploidentical HSCT accompanied by donor lymphocyte infusion (DLI) utilized this approach of MSC co-infusion. In a first report, hematologic recovery and immune reconstitution for NK- and T cells was successful in 5/5 patients and none experienced primary GvHD. The precise contribution of MSCs to these results, however, cannot be assessed since this single-arm study did not contain a control group without MSC co-infusion. MSCs did not prevent a secondary, DLI-induced GvHD, which was, however, accompanied by a favorable GvT effect [82] (Table 2A). In a subsequent study [83] (Table 2B), one of two cohorts did not receive MSC co-transplantation—interestingly, primary HSPC engraftment was equally successful in both cohorts (25/26 patients). Furthermore, no significant difference in the development of grade II-IV GvHD or in event-free survival was found between the two cohorts. These results imply that expected beneficial effects of MSCs (prevention of GvHD and enhancement of hematopoietic engraftment) were minimal in this case. Of note, a favorable GvT effect, as observed in the previous study [82], was in this case insufficient for preventing relapse, which occurred in 75% of the patients. The authors hypothesized that the discrepancy in GvT effects might be due to a high tumor burden in the second study and that induction of remission prior to transplantation is, therefore, important [83]. Another explanation could be a difference in the treatment regimen of allogeneic MSCs in terms of dosage and MSC tissue source, but the latter has not been described in detail by Illhardt et al. [83].

Further evidence for the potential of allogeneic MSCs to support engraftment after allogeneic HSCT and their anti-GvHD effect can be deduced from studies and clinical trials in patients with other malignancies—no beneficial effect of allogeneic MSC co-transplantation on neutrophil- and platelet recovery or GvHD, similar to the result of Illhardt et al. [83], was observed in a pilot clinical trial with pediatric patients of acute leukemia undergoing unrelated UCB transplantation [90] (Table S2C). Two studies with allogeneic BM–MSCs co-transplanted with UCB [91] or BM-/PB-derived HSPCs [92] reported successful prevention of grade III-IV acute GvHD upon MSC co-transplantation, but likewise, no significant effect of MSCs on HSPC engraftment was observed [91] (Table S2D,E). Importantly, the latter study described a significantly increased relapse rate in the MSC-group compared to the control group, despite a comparably low dosage of MSCs [92]. More promising results regarding the improvement of HSPC engraftment were obtained in three clinical studies investigating the use of “off-the-shelf”, UC-derived MSCs in small cohorts of patients with hematologic diseases undergoing UCB transplantation [93,94,95] (Table S2F–H). Compared to a control group, the patients receiving MSC co-transplantation showed a significantly faster recovery of neutrophil- and platelet counts [94]. In all three studies, no significant effect of GvHD prevention was apparent from MSC co-transplantation [93,94,95]. 

When co-transplanting MSCs, the most common administration route is intravenous injection. An alternative strategy, intra-bone injection, has been studied in the last few years in three clinical trials. These studies (Table S2I–K) are still active and results regarding safety and effectiveness of this approach or the superiority compared to intravenous administration are not available yet. Goto et al. [96] (Table S2K) recently reported that no adverse events related to intra-BM injection of MSCs were observed and therefore assessed this treatment strategy to be safe and feasible. Grade II-IV GvHD did not develop in any of the MSC-treated patients, while it did occur in 50% of the control group. No significant improvement of neutrophil- or platelet recovery could be observed compared to the control group [96]. 

Finally, third-party MSCs can also be of use ex vivo, without being transplanted into the patient [97,98]. They have been shown to stimulate ex vivo expansion of allogeneic HSPCs derived from UCB, which then had a favorable effect on platelet- and neutrophil recovery in patients upon transplantation compared to non-expanded HSPCs [99]. Interestingly, MSC-derived extracellular vesicles (EVs) present an efficient cell-free alternative for enhancement of HSPC ex vivo expansion (Ghebes et al., accepted for publication [100], and recently reviewed by Budgude et al. [101]). Further details regarding the experimental results of each study mentioned in this section as well as other ongoing clinical trials (rows L–N) can be found in Table S2.

### 3.2. Autologous MSCs

Autologous MSCs are not frequently used in clinical settings yet due to potential damage by prior high-dose chemotherapy [102,103,104,105] and treatment delays due to the need for ex vivo expansion, which can be intolerable for patients with aggressive diseases [70]. Two studies in breast cancer [106] and malignant lymphomas [107] have explored co-infusion of autologous MSCs during ASCT. In a phase I/II clinical trial with advanced breast cancer patients, intravenous co-infusion of autologous BM–MSCs was shown to entail rapid neutrophil- and platelet recovery (8 days and 8.5 days, respectively) without any infusion-related immediate or delayed toxicity [106] (Table S2O). Importantly, 4 out of 32 patients had BM metastases at the time of BM aspiration and tumor cells were detected during ex vivo expansion of MSCs. In two of those four patients, the tumor cell number could not be reduced during the expansion protocol and MSCs were not reinfused. In a controlled clinical trial with lymphoma patients, autologous MSCs were co-infused during ASCT and led to an improved early lymphocyte recovery (ELR) compared to standard ASCT without MSCs [107] (Table S2P). The positive effect in this study was especially apparent when the number of reinfused HSPCs or lymphocytes was low—in these cases, MSC co-transplantation improved ELR 2.4- and 1.7-fold, respectively, compared to a group that did not receive MSCs [107]. This is an interesting observation because mobilization and collection of a sufficient amount of HSPCs from PB is a common obstacle in NB treatment (unpublished results from our group; [108]). Additionally, it has been demonstrated in non-human primates that intra-bone co-transplantation of autologous MSCs and HSPCs leads to improved engraftment of HSPCs [109]. 

## 4. Safety and Feasibility of MSC Therapy in NB

### 4.1. Safety of MSC (Co-) Infusion

When applying MSCs as a drug delivery vehicle or to improve HSCT outcome, safety and toxicity are crucial to consider. The studies reviewed here (Table 1 and Table 2, Tables S1 and S2) demonstrate the safety and tolerability of MSC (co-)infusion by means of the absence of infusion-related toxicities, no increase in relapse frequency, and no occurrence of transfusion-related mortality (TRM). Furthermore, a systematic review from 2012 [110] evaluated 36 clinical trials with patients of various clinical conditions, eight of which were randomized control trials, and found no association between MSCs (both autologous and allogeneic) and adverse effects such as infusion-related toxicity, organ system complications, infection, death or malignancy; only an association to transient fever. However, none of these clinical trials included cancer patients. Thus, additional potential adverse effects specific to the tumor context have to be taken into account when discussing the safety of MSC therapy in NB.

### 4.2. Influence of MSCs on Tumor Progression

In the context of various cancers, MSCs have been demonstrated to act tumor-supportive [40,41,42]. In NB, we have described an MSC subpopulation that is specifically present in metastasized BM, for which a tumor-related function is conceivable [105]. In a recent review, we have compiled evidence for tumor-supportive and -suppressive functions of MSCs in NB and other cancer types [44]. Based on these insights, it is important to rule out the risk of unintentionally supporting tumor progression before the clinical application of MSC therapy (Figure 1 ④). Relevant factors influencing whether the MSCs’ effect is tumor-supportive or -suppressive might be dose and timing of MSC infusion [111] and tissue origin of MSCs [112]. A systematic review by Christodoulou et al. revealed that UC–MSCs entail a smaller risk of unintentional tumor support than BM– or AT–MSCs [112]. According to a hypothesis of Klopp et al. [111], injection of MSCs into organisms with existing tumors is more likely to entail tumor-growth inhibition, while co-injection of MSCs and tumor cells bears a higher risk of promoting tumor progression. In that case, the risk of the therapeutic use of MSCs in HSCT and as delivery vehicles for patients with established tumors would be minimal. In line with this hypothesis [111], investigations in animal models found no tumor-aggravating effect of intra-cardially administered allogeneic MSCs on precancerous lesions [113] or when using MSCs as a delivery vehicle for IFN-γ in an NB mouse model [59]. While an in vitro study with MSCs engineered to express IL2 did show conflicting results regarding tumor progression, [67], the tumor-targeted production of antitumor agents in MSCs is likely to overcome any (potential) endogenous tumor-supporting effect of MSCs in vivo. 

The risk of unintentional tumor support in HSCT is especially relevant in the context of autologous, BM-derived MSCs because BM metastases increase the risk of altered, potentially tumor-supportive MSCs [105], and BM aspirations can contain contaminating tumor cells [106]. Allogeneic MSCs, therefore, present a more promising alternative to a tumor-independent graft. In the context of HSCT in NB, there is to date no evidence for adverse effects of MSC therapy on tumor progression, because this analysis was not among the outcome measures of relevant studies [82,83]. In earlier years of HSCT, the standard treatment procedure used to be a full allogeneic BM transplant [114], which naturally entailed a co-transplantation of allogeneic MSCs to the recipient, as opposed to peripheral blood stem cell (PBSC) transplants applied nowadays, no increase in relapse frequency was found between BM- compared to PBSC transplants in a phase III trial with patients of hematologic malignancies [115], suggesting that allogeneic MSCs do not pose a significant risk. However, a clinical trial studying allogeneic MSCs co-transplanted with allogeneic HSCT for hematologic malignancies determined a three-fold increased relapse rate of patients that were co-transplanted with HLA-matched sibling MSCs [92].

One option to diminish the risk of a tumor-supportive role of MSCs is irradiation of MSCs prior to infusion. Low-dose irradiation between 2 Gy and 15 Gy has been shown to diminish the immunosuppressive properties and induce anti-tumoral effects of murine BM–MSCs in vivo, determined by reduced tumor volume and prolonged survival of glioma-bearing mice [116]. MSC irradiation was also applied in studies utilizing MSCs as a delivery vehicle for oncolytic viruses to treat NB [70,71,74], but at a higher dose (30 Gy). Whether this irradiation dose entails similar tumor-suppressive functionalities of MSCs has not been determined. Interestingly, the authors did find the migratory capacity of MSCs to be decreased upon 30-Gy irradiation, but this did not seem to impede the success of virotherapy since irradiated MSCs of responders versus non-responders had similar (reduced) migratory capacities [74]. Another approach of controlling the MSCs’ pro- versus anti-tumorigenic behavior is via their immunomodulatory capacities—by stimulating either toll-like receptor (TLR) 4 or 3, a priming of MSCs toward a pro-inflammatory MSC1- or an immunosuppressive MSC2 subtype, respectively, has been shown to be possible, similar to the TLR-based classification of monocytes into M1- and M2 subsets [117]. A subsequent study in an ovarian cancer xenograft mouse model showed that indeed infusion of human MSC1 subtypes attenuated tumor growth, while MSC2 subtypes increased tumor growth [118].

### 4.3. Cell-Free Approach Using Extracellular Vesicles

An alternative way to minimize the risk of tumor-supportive effects of MSCs and overcome limitations in the biodistribution of systemically applied MSCs is the use of MSC-derived extracellular vesicles (EVs), which partly represent the protein, lipid, and nucleic acid content of their parent cell [119]. Both human and murine MSC–EVs were shown to reverse BM radiation damage in a mouse model [120] and human MSC–EVs successfully reached radiation-damaged BM [121] and areas of acute kidney injury [122] in mice. Furthermore, EVs of TRAIL-engineered MSCs showed anti-tumor activity in a melanoma mouse model [52] and EVs obtained from different cell types were shown to function as potential delivery vehicles for oncolytic viruses [123,124]. One case study has also proven the feasibility of applying MSC-derived EVs in humans—a patient suffering from GvHD had no severe side effects of the EV treatment, and GvHD symptoms were significantly improved within two weeks [125].

### 4.4. Influence of Administration Route on MSC Migration

The biodistribution of MSCs is a crucial aspect when assessing the feasibility of their therapeutic use. In animal studies, MSCs are often injected intraperitoneally, subcutaneously, or even intratumorally, which yields superior homing efficiencies than intravenous administration [57,126,127]. While a few clinical trials in adult tumors also apply intraperitoneal (Table S1B,D) or intratumoral (Table S1A) administration of MSCs, the most feasible route in pediatric patients is a systemic administration through the vascular system. In the case of intravenous administration, a major part of MSCs gets entrapped in the lungs and only a small percentage reaches the actual target tissue [48,128,129]. This might be caused by the MSCs’ size and nuclear shape after ex vivo expansion [130,131], and their interaction with adhesion molecules in different tissues [132]. Intra-arterial injection diminishes this first-pass effect but bears the risk of microvascular occlusions [130,133]. Interestingly, intra-bone co-transplantation of MSCs was shown to enhance HSPC engraftment, even at BM sites distant to the site of transplantation, in non-human primates [109]; an administration route that is also clinically evaluated in cancer patients [96] (Table S2I–K). 

Notably, MSCs are often undetectable after systemic administration but, nevertheless, immunomodulatory effects can be observed. This suggests that the MSCs’ immunomodulatory function is mediated by an indirect mechanism, for example via the polarization of macrophages toward the immunosuppressive M2 subset by MSC-derived factors such as indoleamine 2,3-dioxygenase (IDO) and chemokine ligand 2 (CCL2), and/or by phagocytic uptake of MSC debris after induced apoptosis [48,134,135,136] (Figure 1 ⑤).

### 4.5. MSC Engraftment in the BM after HSCT

Research results obtained in mouse models confirm the ability of MSCs to migrate to wounded microenvironments [36] and radiation-damaged tissue [137,138]. However, both autologous and allogeneic MSCs might not permanently engraft in the BM [84,91,96,139,140]. In many HSCT studies, the level of MSC engraftment is not assessed, and it can be difficult to do so due to restrictions in sampling options and detection methods [90]. Interestingly, exceptions with successful engraftment are seen, for example, upon allogeneic BM transplantation in the treatment of osteogenesis imperfecta (OI) [141] and in allogeneic MSC transplantation in the treatment of severe acute anemia (SAA) [142], both of which resulted in successful, albeit low, MSC engraftment levels for up to three months. Leibacher et al. suggested that allogeneic MSC engraftment might depend on the existence of “empty niches” [143], which are created in the context of OI and SAA due to stromal/BM defects underlying those diseases. In the context of HSCT, this condition might be met when parts of the stromal compartment have been affected/eliminated by MAT. A comparison of high-dose chemotherapy-treated and untreated BM of patients with hematologic malignancies indeed demonstrated a functional impairment of the stromal compartment [102,103]. Similarly, MSCs were shown to be highly sensitive to the chemotherapy compound paclitaxel [104] and to be reduced in quantity after induction therapy in NB patients [105]. Another factor influencing engraftment could be the dose of MSCs, with higher doses increasing the chance for successful engraftment [142].

However, even if MSCs do not engraft (or are at least not proven to engraft), they exert beneficial functions nevertheless, as demonstrated in the above-mentioned studies and trials (Table 2; Table S2), suggesting that even a temporary presence of donor MSCs has some effects and/or that indirect mechanisms via other cell types are involved, as discussed in the previous paragraph. The latter effect would make permanent MSC engraftment redundant and explain the, often impossible, post-transplantation detection of MSCs. 

### 4.6. Tumor-Tropism of MSCs upon Use as Delivery Vehicles 

Similarly, the question arises whether therapeutically engineered MSC products reach NB tumor sites effectively for the delivery of anti-cancer agents (Figure 1 ④). The tumor-tropism of MSCs from different sources has been studied in animal models and one patient study (an overview is shown in Table 3 and Table S3). The success of tumor homing depends greatly on the administration route (as discussed above) and likely on other factors such as tissue origin, surface molecule expression, cell cycle phase, and passage number [21,144,145]. Tracking of MSC migration is facilitated in animal models for example by firefly luciferase-labeled cells [36] but presents a challenge in human patients in terms of tissue penetration, GMP standards, and costs. Non-invasive options for cell tracking with clinical potential (involving e.g., MRI/PET scans) have been reviewed by Hong et al. [146]. There is to date no literature available from clinical trials examining the tumor-tropism of MSCs in NB patients. A phase I clinical study with prostate cancer patients aiming to investigate specifically the tumor-tropism of allogeneic MSCs infused the MSCs prior to prostatectomy, which allowed for assessment of MSC presence in the resected tumor tissue. No graft DNA was detectable in the tumor tissue of all seven patients, and the study was prematurely terminated [147]. A therapeutic effect of MSC-delivered anti-cancer agents and oncolytic viruses, however (see Table 1 and Table S1) suggests at least a transient presence of MSCs at the tumor site [59,70]. Homing efficiencies could be improved by modifying MSCs through genetic engineering or cell surface modifications prior to transplantation (reviewed by Krueger et al. [148]). For example, modifying MSCs to induce/increase the expression of adhesion molecules such as CCR1 and CXCR1 might improve their tumor-homing efficiency, as suggested by studies in glioma [149] and with oncolytic virus-loaded MSCs in NB [74]. This effect might be evoked by improved chemotaxis towards the source of their respective ligands, CCL5 and IL8, both of which are produced by (NB) tumors and/or the tumor microenvironment [40,150,151]. Furthermore, the migratory capacity of MSCs was shown to be influenced by the cell cycle [21], which suggests that the selection of cells in the G1 phase or experimental modulation of the cell cycle could entail improved migration efficiencies. 

For NB and other malignancies that arise in or metastasize to the BM, specific targeting of this secluded niche is especially crucial. Strategies currently under development are extensively reviewed by Mu et al. [152] and include but are not limited to (i) drug-carrying liposomes and micro-/nanoparticles, which are phagocytosed by BM macrophages or (ii) which are modified to bind with high affinity to the bone mineral; furthermore (iii) antibody-conjugated immunocytokines such as IL2 can be targeted to antigens of the neoangiogenic tumor vasculature in the BM, an approach currently evaluated in a phase I/II trial for solid tumors [153]. Whether it is possible to similarly apply such approaches to modify MSCs (or MSC-derived exosomes) as the drug carrier to enhance BM-tropism remains to be elucidated. Alternatively, MSC targeting to the BM could be achieved by overexpression of CXCR4, which has been demonstrated in mouse models to specifically enhance migration and engraftment of AT–MSCs into the CXCL12-abundant BM [154,155]. Another interesting approach is to convert the MSC cell surface glycoprotein CD44 into its sialofucosylated glycoform, called hematopoietic cell E-selectin/L-selectin ligand (HCELL), which then binds to vascular E-selectin within specialized BM vasculature and improves BM infiltration [156]. 

### 4.7. Influence of Ex Vivo Expansion of MSCs

One obstacle when considering the therapeutic use of MSCs is the need for cultural expansion to reach the target therapeutic dose (Figure 1 ①), which typically ranges around 1–2 × 10^6^ cells per kilogram body weight, and becomes especially critical with increasing MSC donor age [157]. The majority of data on the clinical use of MSCs is thus obtained based on ex vivo expanded MSCs. Even though early experiments in mouse models found a similar effect of non-expanded MSCs on HSPC engraftment compared to that of culture-expanded MSCs [158], plastic adherence during ex vivo expansion and the culture medium used might change the phenotype and functionality of these MSCs [159,160,161]. For example, altered Wnt signaling [98], adhesion molecule expression, and size of MSCs after culture expansion have been observed [130,133], which might lead to lung entrapment and the risk of microvascular occlusions after intravascular injection (Figure 1 ②). Supporting these considerations, primary murine MSCs have been shown to lose their BM homing ability following culture [144]. Similarly, niche damage repair, intra-bone HSPC transplantation efficacy, and long-term engraftment of primary BM–MSCs have been shown to be superior to their ex vivo expanded counterparts [162]. An interesting approach to circumvent the adverse effects of ex vivo expansion is the reprogramming of MSCs into a “revitalized” state using specific transcription factors [163]. These modified MSCs have been shown to regain their hematopoietic support function, as determined by improved repopulation capacity upon transplantation of HSPCs co-cultured with these MSCs into a mouse model. 

## 5. Future Directions and Concluding Remarks

An overview of the current applications of MSCs in tumor therapy and associated risks and challenges is given in Figure 1. Current limitations when using MSCs as delivery vehicles for anti-cancer agents include the low bioavailability after systemic administration. In NB, targeting not only the primary tumor but especially the BM metastases is of critical importance to effectively eliminate the minimal residual disease, which is the main cause for relapse [164]. This has successfully been shown in one NB patient in a study with oncolytic virus-loaded MSCs (“CELYVIR”) [71] and raises hope that such BM targeting is feasible. Various ways of improving tumor-tropism of MSCs have been suggested, including genetic engineering [50,156] or radiation of MSCs [165] to enhance their migration to the BM or tumor site. 

In the context of HSCT, MSC co-transplantation can have beneficial effects. Homing to the BM and engraftment therein need to be studied in detail in order to understand the mode of action and the resulting consequences on treatment outcome. Indirect, immunomodulatory effects on other immune cells, independent of successful MSC engraftment, have been suggested to be responsible for the beneficial effects of MSCs, especially in preventing GvHD upon intravenous injection [48]. 

For both the delivery of anti-cancer agents and co-transplantation in HSCT, potential tumor-supportive effects of MSCs remain the biggest risk, especially in the case of BM-derived autologous MSCs in NB. Irradiating MSCs prior to infusion [70,71,74,116] and MSC-derived EVs as a cell-free alternative [52,120,121,122,123,124,125] could reduce the risk of unintentionally supporting tumor progression. The use of EVs furthermore presents a potential alternative to provide the beneficial effects of MSCs to the BM or tumor site [121].

In conclusion, the interest in applying MSCs as an adjuvant to cancer therapy has risen fast in the last years due to scientific successes in preclinical in vitro and animal model studies. Especially for high-risk NB patients, who have a poor prognosis despite an intense multi-modal treatment, oncolytic virotherapy might be a valuable addition to conventional therapy. MSC-mediated delivery of other anti-cancer agents in the NB context, however, is currently only evaluated in preclinical studies and requires further research before it can be safely applied in the clinic. Similarly, co-transplantation of allogeneic MSCs during HSCT has barely been studied in NB patients and requires further evaluation in larger controlled clinical trials. 

## Figures and Tables

**Table 1:** Overview of studies and clinical trials (registered on clinicaltrials.gov; accessed on 20 January 2021) investigating the potential use of MSCs as delivery vehicles for oncolytic viruses in neuroblastoma.

	Phase	Anti-Cancer Agent	Properties and Dose of MSCs	Administration Route	Nr. of Patients	Disease Context	Key Findings	Publication/Status	ClinicalTrials.gov Identifier	Year
	**Delivery of Oncolytic Viruses in MSCs**									
A	n/a	ICOVIR-5, an oncolytic adenovirus	autologous irradiated BM-MSCs (“CELYVIR”), 2–4 doses of each 0.1–0.9 × 10^6^ cells/kg	“infused through a central line”	*n* = 4, single-arm study	Therapy-resistant NB patients	CR (>3 years) in 1 out of 4 patients, virus detected in BM biopsy	García-Castro et al. [71]	n/a (exploratory study)	2010
							Very low systemic toxicity			
B	n/a	ICOVIR-5, an oncolytic adenovirus	autologous irradiated BM-MSCs (“CELYVIR”), 4–70 doses of each 150–2640 × 10^6^ cells	“systemic infusion”	*n* = 12, single-arm study	NB	In vitro assays: adhesion molecules like CXCR1 and CCR1 significantly higher in MSCs of responders	Melen et al. [74]	n/a (compassionate use program)	2016
							Mild and auto-limited virus-related toxicities; none had grade 3+ toxicities			
							Clinical response (SD, PR, CR) in 5 out of 12 patients			
C	I/II	ICOVIR-5, an oncolytic adenovirus	autologous irradiated BM-MSCs (“CELYVIR”), 2 × 10^6^ cells/kg (children) or 0.5–1 × 10^6^ cells/kg (adults)	intra-venous	*n* = 9 (pediatric), *n* = 7 (adult); single-arm study	Metastatic and refractory tumors, including NB	Adenoviral replication detected by PCR in 7 out of 9 pediatric patients but in none of the adults	Ruano et al. [70]	NCT01844661	2013
							SD in 2 out of 4 patients			
							Increasingly higher numbers of circulating lymphocytes (B and T) in responders compared to non-responders			
							No grade 2–5 toxicities were reported.			

IL12–Interleukin-12, MTD–maximum tolerable dose, DLT–dose-limiting toxicities, OS–overall survival, SD–stable disease, PR–partial response, CR–complete response.

**Table 2:** Overview of clinical trials registered on clinicaltrials.gov; accessed on 20 January 2021 investigating a potential benefit of MSCs in HSCT in neuroblastoma.

	Phase	Details	Properties and Median Dose of MSCs	Nr. of Patients	Disease Context	Key Findings	Publication/Status	ClinicalTrials.gov Identifier	Year
**allo-MSCs**									
A	I	Allogeneic MSCs co-transplanted with haplo-HSCT and subsequent DLI	Allogeneic BM-MSCs, 0.75 × 10^6^ MSC/kg	*n* = 5 (all received MSCs, no control)	NB (relapsed/refractory)	2 of 5 patients achieved long-lasting remission (40 and 42 months)	Toporski et al. [82]	NCT00790413	2008
						Neutrophil recovery in all children (median 13 days), platelet recovery in 4/5 children (12 days)			
						Rapid immune reconstitution of NK- and T cells			
						No primary aGVHD, but 4/4 patients had secondary GvHD after DLI			
B	I	Allogeneic MSCs co-transplanted with haplo-HSCT and DLI	Allogeneic MSCs, no details or dose mentioned	MSC(+): *n* = 9, MSC(-): *n* = 17	NB (relapsed/refractory)	Primary engraftment in 96% (25/26) of the patients	Illhardt et al. [83]	NCT00790413	2018
						GvHD: no significant differences between MSC and non-MSC group			

DLI–donor lymphocyte infusion, haplo-HSCT–haploidentical HSCT, aGvHD–acute graft-versus-host disease.

**Table 3:** Overview of studies investigating tumor-tropism of MSCs in neuroblastoma.

Model	MSC Origin	Labeling Method	Administration Route	Maximum Follow up	Detection in Tumor	Publication
NB xenograft model in NOD/SCID mice	human, BM	Radiolabeling	IP	48 h	Yes	Cussó et al. [126]
TH-MYCN transgenic mouse	human, AT	Near-IR	IP, IV	24 h	Only i.p.	Kimura et al. [127]
TH-MYCN transgenic mouse	mouse, BM	GFP	IP	2 weeks	Yes	Maniwa et al. [57]

IP–intraperitoneally; IV–intravenously, AT–adipose tissue, BM–bone marrow.

## Supplementary Materials

The following are available online at https://www.mdpi.com/2075-4426/11/3/161/s1, Table S1: Overview of clinical trials registered on clinicaltrials.gov (accessed on 20 January 2021) investigating the potential use of MSCs as delivery vehicles for anti-cancer agents and oncolytic viruses in other solid tumors, Table S2: Overview of studies and clinical trials registered on clinicaltrials.gov (accessed on 20 January 2021) investigating a potential benefit of MSCs in HSCT in context of hematological diseases and cancer. Table S3: Overview of studies investigating tumor-tropism of MSCs in other solid tumors.
